# Photoelectric Characteristics of a Large-Area n-MoS_2_/p-Si Heterojunction Structure Formed through Sulfurization Process

**DOI:** 10.3390/s20247340

**Published:** 2020-12-21

**Authors:** Yoonsok Kim, Taeyoung Kim, Eun Kyu Kim

**Affiliations:** Department of Physics and Research Institute of Natural Science, Hanyang University, Seoul 04763, Korea; francis1206@hanyang.ac.kr (Y.K.); brownmunn@hanyang.ac.kr (T.K.)

**Keywords:** MoS_2_, transition metal dichalcogenide, heterojunction, photodiode

## Abstract

Two-dimensional (2D) materials, such as molybdenum disulfide (MoS_2_) of the transition metal dichalcogenides family, are widely investigated because of their outstanding electrical and optical properties. However, not much of the 2D materials research completed to date has covered large-area structures comprised of high-quality heterojunction diodes. We fabricated a large-area n-MoS_2_/p-Si heterojunction structure by sulfurization of MoO_x_ film, which is thermally evaporated on p-type silicon substrate. The n-MoS_2_/p-Si structure possessed excellent diode characteristics such as ideality factor of 1.53 and rectification ratio in excess of 10^4^. Photoresponsivity and detectivity of the diode showed up to 475 mA/W and 6.5 × 10^11^ Jones, respectively, in wavelength ranges from visible to near-infrared. The device appeared also the maximum external quantum efficiency of 72%. The rise and decay times of optical transient response were measured about 19.78 ms and 0.99 ms, respectively. These results suggest that the sulfurization process for large-area 2D heterojunction with MoS_2_ can be applicable to next-generation electronic and optoelectronic devices.

## 1. Introduction

Two-dimensional (2D) materials have been intensely studied due to their extraordinary electrical and optical properties ever since graphene was first mechanically exfoliated from graphite by using Scotch tape [1]. Because of weak van der Waals force between each layer in the layered structure, 2D materials can be easily exfoliated to thin film. However, the zero bandgap of graphene obstructs its application for semiconductors in electronic and optoelectronic operation [2,3]. Therefore, transition metal dichalcogenides (TMDs) have aroused significant interest due to their appropriate band gap for applications such as field-effect transistors (FETs) [4,5,6,7], logic circuits [8,9,10,11], junctions [12,13,14,15], photodetectors [16,17,18], memory devices [19,20,21], flexible devices [22,23], and sensors [24,25]. The TMDs consist of one transition metal and two chalcogenides formed into a sandwich structure monolayer. Molybdenum disulfide (MoS_2_), a typical representative of the TMDs, possesses a direct band gap of 1.8–1.9 eV in a monolayer, and an indirect band gap of 1.2–1.3 eV in multilayer forms [26]. MoS_2_ shows n-type behavior and is commonly used as semiconducting layer. Moreover, MoS_2_ has a high carrier mobility sufficient to apply it in electronic devices [27,28]. 

Recent works on MoS_2_ and other 2D materials-based optoelectronic and photo-detecting devices includes a heterojunction fabricated by vertically stacked MoS_2_ and WSe_2_ which showed photodiode properties [29]. MoS_2_ optoelectronic performance from a homojunction formed by chemical doping [30] and MoS_2_ phototransistors with high carrier mobility were studied also in wavelength ranges from ultraviolet to infrared [31]. Moreover, photodiodes using MoS_2_ and other conventional semiconductor materials such as p-type silicon and GaN have been studied for years [32,33,34]. The samples in most of these research were used exfoliated flakes by mechanical exfoliation, however, which the limited size of flakes means difficulty using them in practical applications. Some research has been demonstrated using large-area MoS_2_ with other conventional semiconductors, but progress on large-area synthesis of MoS_2_ film for optoelectronic devices has been limited. For practical applications to develop, a large-area uniform growth technique is essentially required. 

In this study, we synthesized large-scale area MoS_2_ films on p-type silicon substrates through a sulfurization process of substoichiometric molybdenum trioxide (MoO_x_) layer. To compare the physical properties of pure and sulfurized MoO_x_ films, atomic force microscopy (AFM), X-ray diffraction (XRD), Raman spectroscopy, and X-ray photoelectron spectroscopy (XPS) were measured. The electrical and optoelectronic characteristics of n-MoS_2_/p-Si heterojunction diode were analyzed by I-V measurements with light illumination.

## 2. Experimental Details

(100)-oriented p-type silicon substrates were used to fabricate the p-n junction with large-area MoS_2_. Resistivity of the substrate was 1.7 Ω·cm measured by a Hall measurement system (HMS-5000, Ecopia, Anyang, Korea). This value corresponds to the doping concentration of 8.52 × 10^15^ cm^−3^. Buffered oxide etchant was used to remove any native oxide layer on the silicon surface prior to ultrasonic cleaning in acetone, methanol, isopropanol, and de-ionized water for 10 min each to remove any contaminants. Substrates were then dried by blowing N_2_ gas. The formation processes of n-MoS_2_/p-Si heterojunction structures are as follows; At first, by using a fully stoichiometric molybdenum trioxide (MoO_3_, CERAC Inc., Milwaukee, WI, USA) powder, the MoO_x_ thin films were deposited on p-type silicon substrates by using thermal evaporation technique. Here, the evaporation chamber was kept at ~8 × 10^−4^ Pa. In this process, films square of dimension 5 × 5 mm^2^ were obtained using shadow mask. Next, the large-area MoS_2_ thin film was chemically synthesized by sulfurization process in a quartz tube. An alumina boat was filled with 0.4 g of sulfur (iTASCO Inc., Seoul, Korea) powder. When the sulfurization process was proceeded, the furnace was heated from room temperature to 850 °C. 100 sccm of argon gas flow was used to transport the sulfur vapor. The furnace was maintained at 850 °C for 30 min under 1.0 × 10^5^ Pa. After 30 min, the furnace was cooled down to room temperature.

To compare the physical properties of pure and sulfurized MoO_x_ films, AFM (XE-100, Park Systems, Seoul, Korea), Raman spectroscopy (NRS-3100, JASCO, Tokyo, Japan), XRD (SmartLab, Rigaku, Tokyo, Japan), and XPS (K-Alpha+, Thermo Fisher Scientific, Waltham, MA, USA) were measured. For n-MoS_2_/p-Si diode, Ti/Au (5 nm/50 nm) electrode as an ohmic contact to the MoS_2_ film was patterned through shadow mask and thermally evaporated on the MoS_2_ film. Indium was also used to contact p-type silicon. Finally, Au/Ti/n-MoS_2_/p-Si/In structure device was fabricated. The electrical characterization was performed by a semiconductor parameter analyzer (HP-4156A, Agilent, Santa Clara, CA, USA) with and without light illumination.

## 3. Results and Discussion

**Figure 1:**
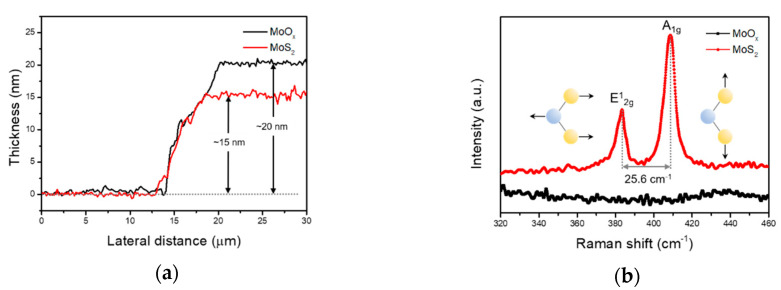
(**a**) Thickness of the MoO_x_ and MoS_2_ films measured by AFM are about 20 nm and 15 nm, respectively. (**b**) Comparison of the MoO_x_ and MoS_2_ films from Raman spectroscopy. The sulfurized film shows two typical phonon modes, E^1^_2g_ and A_1g_, with peak difference of 25.6 cm^−1^.

The MoS_2_ thin film was synthesized on upper side of the p-type silicon substrate by sulfurization process. The junction of the MoS_2_ film and p-type silicon substrate acts as a heterojunction diode consisting of two different semiconductors. The thickness profile measured by AFM is shown in Figure 1a. The thickness of MoO_x_ film was confirmed 20 nm, while that of MoS_2_ film was 15 nm, corresponding to bulk MoS_2_. Figure 1b shows Raman spectroscopy of the MoO_x_ and MoS_2_ films. Full range measurement is also shown in Figure S1. In case of MoO_x_ film, any peaks of Raman spectra were not measured. Because MoO_x_ film deposited by thermal evaporation is amorphous state [35]. While two typical phonon vibration modes were observed on the sulfurized MoS_2_ film, E^1^_2g_ and A_1g_ phonon vibration mode. The E^1^_2g_ mode indicates an in-plane vibration of Mo and S atoms, whereas A_1g_ mode represents an out-plane vibration, as shown in the inset of Figure 1b. Each of the phonon vibration modes is extracted at 382.7 cm^−1^ and 408.3 cm^−1^ by Gaussian curve fitting. The difference of the two phonon vibration modes is calculated to be 25.6 cm^−1^.

According to a previous report and AFM measurement of this work, it is confirmed that the MoS_2_ film is consistent with the bulk MoS_2_ [36]. Additionally, the sulfurized film shows good homogeneity from the Raman data in Figure S2 which is measured randomly at three points, two edges and one center parts. To verify the structure and crystallinity of the films, Raman spectroscopy and XRD measurement were performed on the p-type silicon substrates. 

**Figure 2:**
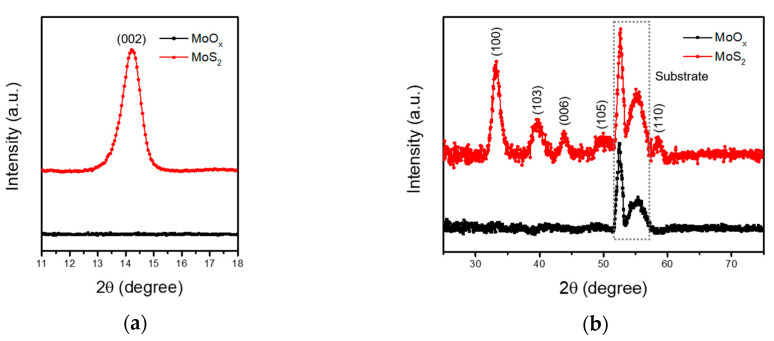

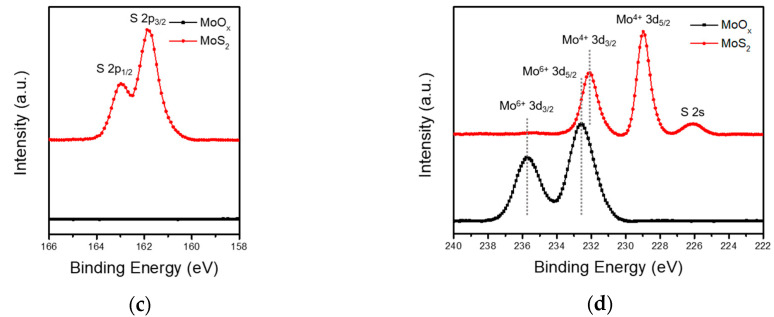
(**a**) (002)-Oriented XRD data, (**b**) (100), (103), (006), (105), and (110)-oriented XRD spectra. The gray-colored dash square indicates the substrate peaks. XPS spectra of Mo 3d (**c**) and S 2p (**d**) core levels of the MoO_x_ and MoS_2_ films.

Figure 2a,b display the XRD data of MoO_x_ and MoS_2_ films. The thermally evaporated MoO_x_ film showed only silicon substrate peaks as reported previously [35]. This demonstrates that the MoO_x_ film exists in an amorphous state without crystallinity. After sulfurization process, the MoS_2_ related XRD data clearly showed diffraction peaks indexed to (002), (100), (103), (006), (105), and (110) of hexagonal phase MoS_2_ (JCPDS No. 47-1492). It is clear that the sulfurized MoS_2_ film possesses high crystallinity. From the XRD data, average grain size was calculated to be about 10 nm using Scherrer equation as shown in Figure S3. XPS measurement was performed to analyze the chemical bonding of the MoO_x_ and MoS_2_ films. Figure 2c shows the S 2p_1/2_ and S 2p_3/2_ peaks at 163.0 eV and 161.8 eV, respectively, of MoS_2_ film. No S peaks exist from MoO_x_ film. Figure 2d displays the two distinct peaks for 3d electrons of the Mo atoms and one peak for 2s electrons of the S atoms. The MoO_x_ film showed peaks for Mo^6+^ 3d_3/2_ and Mo^6+^ 3d_5/2_ at 235.7 eV and 232.6 eV, respectively. These peaks are consistent with those of MoO_3_. Additionally, no such peaks were observed related to S atoms. On the other hand, the sulfurized MoS_2_ film showed peaks at 232.1 eV, 229.0 eV and 226.1 eV which correspond to Mo^4+^ 3d_3/2_, Mo^4+^ 3d_5/2_ and S 2s, respectively. The XPS spectra results are consistent with those of the pristine sample. The O 1s core level and the atomic ratio are shown in Figure S4.

**Figure 3:**
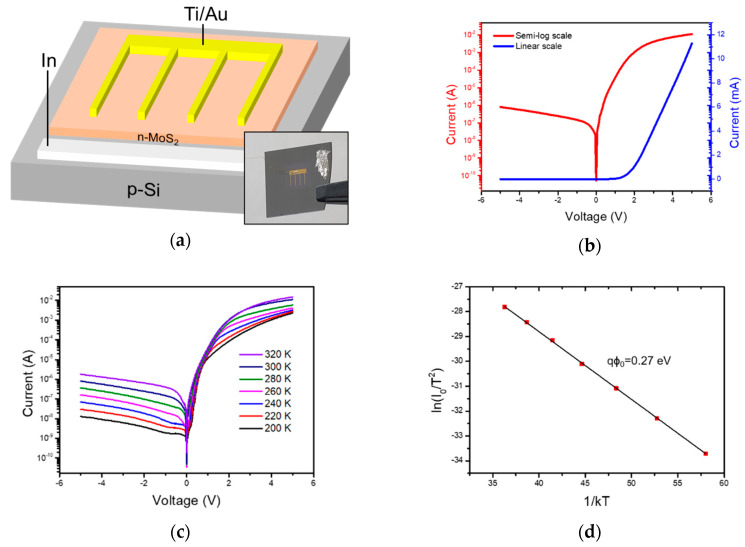
(**a**) Schematic diagram of the n-MoS_2_/p-Si heterojunction diode. The inset in (**a**) shows photograph of the heterojunction diode. (**b**) Electrical characterization of n-MoS_2_/p-Si heterojunction on semi-log scale (red) and linear scale (blue). (**c**) Temperature dependent I-V measurement in semi-log scale. (**d**) Richardson plot from the I-V measurement. The barrier height was calculated to be 0.27 eV.

Figure 3a displays a schematic diagram of the n-MoS_2_/p-Si heterojunction diode. For Au/Ti/n-MoS_2_/p-Si/In structure, the current versus voltage (I-V) characteristics of the diode measured under dark conditions are shown in Figure 3b. The red line indicates the semi-log and the blue line indicates linear scale of I-V characteristics. The device showed clear rectifying behavior similar to that of p-n diodes. The reverse current was 8.0 × 10^−7^ A at −5 V and the forward current was 1.1 × 10^−2^ A at 5 V. The rectification ratio of the diode was calculated to exceed 10^4^, which is comparable to the previous report from our laboratory [32].

The I-V characteristics of the diode can be analyzed by using the following diode equation:

(1) $$I=I_{0}\left[\exp\left(\frac{\mathrm{qV}}{\mathrm{nkT}}\right)-1\right]$$

where I_0_ is the reverse bias saturation current, q is the electron charge, k is the Boltzmann’s constant, n is the ideality factor of the diode, and T is the absolute temperature. In the voltage range of V ≥ 4kT/q, the ‘−1’ term in the equation can be ignored. Therefore, the ideality factor of the heterojunction can be calculated by the following equation:

(2) $$n=\frac{q}{\mathrm{kT}}\frac{\mathrm{dV}}{d\left(\mathrm{lnI}\right)}$$

The ideality factor is an important value that provides an estimate of how closely the fabricated junction approaches the ideal diode situation. The ideality factor of the n-MoS_2_/p-Si diode was calculated to be 1.53, which indicates that the carrier transport phenomenon originated from both ideal diffusion process and recombination process. This value was calculated in the range of satisfying not only V ≥ 4kT/q mentioned previously, but also at low voltage enough to neglect series resistance and high injection effect. The recombination process may have originated from the existence of interface states of the n-MoS_2_/p-Si junction. Next, we further performed temperature dependent I-V measurements to calculate the effective barrier height of the diode. Figure 3c shows the temperature dependent I-V characteristics of the heterojunction. As the temperature decreases, the current value decreases. The barrier height can be determined by Richardson plot which is evaluated from thermionic emission theory of charge carriers:

(3) $$\ln\left(\frac{I_{0}}{T^{2}}\right)=\ln\left({\mathrm{AA}}^{*}\right)-\frac{q\phi_{0}}{kT}$$

Here, A is the junction area (25 mm^2^) of the device, A* is the effective Richardson constant, ϕ_0_ is the barrier height of the device, k is Boltzmann’s constant, and T is the absolute temperature. This calculation was carried out in the temperature range of 200–320 K. From the Richardson plot as shown in Figure 3d, the barrier height of 0.27 eV was extracted for the n-MoS_2_/p-Si heterojunction.

**Figure 4:**
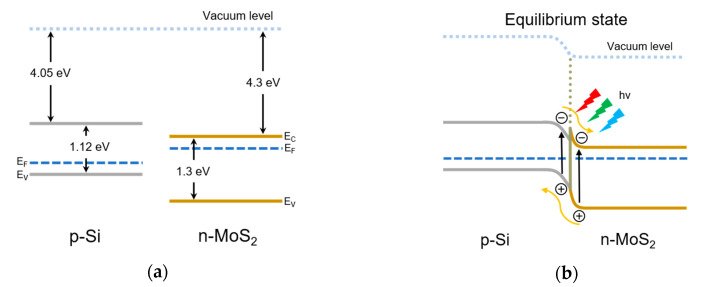
(**a**) p-type silicon and n-MoS_2_ energy band diagrams. (**b**) The energy band diagram of n-MoS_2_/p-Si heterojunction in equilibrium state.

From the energy band diagram in Figure 4, the band offset of the n-MoS_2_/p-Si diode is confirmed as a type Ⅱ heterojunction. The electron affinity and band gap of bulk MoS_2_ are 4.3 eV and 1.3 eV, respectively [37]. In the case of silicon, electron affinity and band gap are 4.05 eV and 1.12 eV, respectively. Due to the energy difference of Fermi levels, energy levels bend each other as the junction is formed. At the equilibrium state, the electrons in the n-type MoS_2_ lay er diffuse to the p-type silicon layer, while the holes in the p-type silicon diffuse to the n-type MoS_2_. A depletion layer is thereby created at the interface of n-MoS_2_/p-Si heterojunction.

A certain barrier height can be created, making it possible to show rectification characteristics. As the forward bias is applied, the barrier height and the depletion region decrease. Therefore, the carriers drift to each of the semiconductor layers. On the other hand, as the reverse bias is applied, the barrier height and the depletion region increase. The carriers cannot then drift to each of the semiconductor layers. Under illumination, electron-hole pairs will be generated and separated by the electric field in the depletion region and with applied bias. Photocurrent increases by the photo-generated carriers. On the reverse bias, the large electric field can serve to separate the photo-generated carriers and the photocurrent can be enhanced.

**Figure 5:**
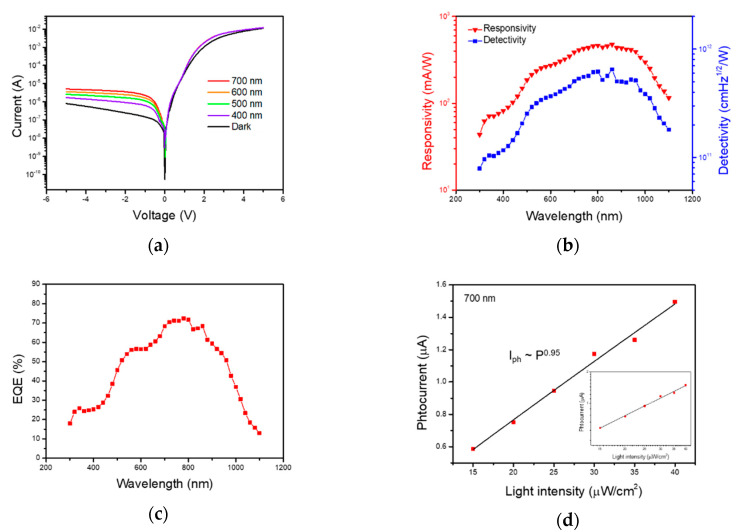
(**a**) I-V measurement comparison at the different visible wavelengths and light intensity of 50 μW/cm^2^. (**b**) Wavelength dependent responsivity, detectivity, and (**c**) EQE. (**d**) Light intensity versus photocurrent graph in linear scale and semi-log scale (inset).

We further investigated the photo-response characteristics under light illumination. Figure 5a describes the I-V characteristics while varying the illumination wavelength from 400 nm to 700 nm at increments of 100 nm, with constant light intensity of 50 μW/cm^2^. At the forward bias region, only small current variation occurred compared with the dark condition. However, because of large electric field at the reverse bias region, a significant current difference appeared of about 10 times larger than the dark current. In this range, maximum photocurrent was generated around 700 nm. Furthermore, we measured the photo-response characteristics more precisely at a wide spectral range of wavelengths. The photoresponsivity represents the response capability of generated photocurrent per incident light, which is also called spectral responsivity.

The left axis of Figure 5b shows the photoresponsivity of the device at fixed reverse bias voltage of −5 V. It can be calculated from the following equation:

(4) $$R=\frac{I_{\mathrm{ph}}}{P\times A}$$

Here, R is the photoresponsivity (A/W), P is the light intensity (W/cm^2^), A is the active junction area (25 mm^2^), I_ph_ is photocurrent which can be calculated as I_light_-I_dark_ (I_light_: current at light illumination, I_dark_: current at dark condition). The n-MoS_2_/p-Si heterojunction is confirmed as possessing broadband photoresponsivity at spectral range from about 300 nm to 1100 nm. The photoresponsivity increases with increasing wavelength to 860 nm, which corresponds to I-V characteristics under illumination. At longer wavelengths, photoresponsivity decreases. This tendency is like other previously reported results [14,37,38]. The maximum photoresponsivity of the n-MoS_2_/p-Si heterojunction shows 475 mA/W at 860 nm light. The photoresponsivity value of our n-MoS_2_/p-Si device is lower than that of mechanically exfoliated single crystal MoS_2_ based photodiode but comparable to previous reports [14,37].

Next, we evaluated the detectivity at reverse bias of −2 V. The detectivity describes the capacity for photo-detecting at weak optical power, which can be calculated using the following equation:

(5) $$D^{*}=\frac{A^{1/2}R}{{\left(2{\mathrm{qI}}_{\mathrm{dark}}\right)}^{1/2}}$$

where D^*^ is the detectivity. To show high detectivity, low dark current and high responsivity are needed. Based on the above equation, the detectivity is changed from 7.9 × 10^10^ Jones to 6.5 × 10^11^ Jones as shown in right axis of Figure 5b. The maximum detectivity of 6.5 × 10^11^ Jones is measured at the wavelength of 860 nm. Because of the low dark current at the reversed bias, which is a typical characteristic of junction type photodetectors, the calculated detectivity is much higher than phototransistors or photoconductors [39]. The external quantum efficiency (EQE) is defined as the number of photogenerated carriers produced per illuminated photons. This can be calculated by using the following equation:

(6) $$\mathrm{EQE}\;\left(\%\right)=\frac{\mathrm{electrons}/s}{\mathrm{photons}/s}\times 100=\frac{I_{\mathrm{ph}}}{q}\left(\frac{h\nu }{P_{\mathrm{opt}}}\right)\times 100$$

Here, h is Planck’s constant, ν is the frequency of the light, and P_opt_ is the optical power. An EQE value of 100% would show the ideal case in which one photon generated one electron-hole pair. Figure 5c shows the measured EQE of our device at reverse bias of −5 V. In our n-MoS_2_/p-Si device, the maximum EQE extracted was 72% at 780 nm wavelength.

We also extracted photo-response properties under various light intensities. The photocurrent was measured by varying the light intensity from 15 to 40 μW/cm^2^ at wavelength of 700 nm. The photocurrent data in Figure 5d and the inset display not a linear shape but rather a power-law fitting shape as a function of light intensity. It can be described by the following equation:

(7) $$I_{\mathrm{ph}}={\mathrm{AP}}^{\alpha }$$

where A is a scaling constant, P is the light intensity, and α is an exponent determining factor. The exponent value is less than unity because of complex process of electron-hole generation, trapping, and recombination [37,38,40]. The exponent α of our n-MoS_2_/p-Si diode was calculated to be 0.95 at a reverse bias of −1 V, which means that the photocurrent value is close to the linear graph and the n-MoS_2_/p-Si diode has a remarkable capability for photodetection with a low density of defect trap states. Since silicon substrate is a perfect single crystal without defect, the defect trap states may originate from the MoS_2_ film. Previous paper reported the trap states of MoS_2_ films were sulfur vacancies and antisite defects [41]. Moreover, sulfur vacancies were observed in the sputtered film previously [42,43].

The light illumination response time is another key factor for fast light detecting application. We evaluated the response speed of the n-MoS_2_/p-Si diode as a photodetector at a fixed reverse bias voltage of −1 V under square-pulse light illumination with a 780 nm wavelength. Figure S5a shows the photocurrent transient characteristics for three cycles which are measured at 1 Hz by pulse generator. The results demonstrate excellent repeatability and stability. We calculated the response time and decay time of the n-MoS_2_/p-Si diode with exponential fitting. The rising part and decaying part are displayed in Figure S5b and Figure S5c, respectively. The rise time τ_r_ and the decay time τ_d_ were extracted to be 19.78 ms and 0.99 ms, respectively. These results are faster than silicon device which indicates that the photo-generated electron-hole pairs are effectively generated and separated by electric field and reverse bias of the junction interface. Our n-MoS_2_/p-Si device and the previously investigated MoS_2_- and Si-based photodetectors are compared in Table 1. Our device displays inferior photoresponsivity compared to the MoS_2_/p+-Si photodiode, which was fabricated via a mechanical exfoliation method in our lab. However, the detectivity of our device is higher, and the rise time and decay time are lower as well [32]. Furthermore, our photodiode showed superior responsivity to previously reported large-area satisfying devices.

## 4. Conclusions

In summary, n-MoS_2_/p-Si heterojunction was fabricated by sulfurization process of the MoO_x_ film, which is thermally deposited on the p-type silicon substrate. Their electrical properties at room temperature typically showed the ideality factor of 1.53 and rectification ratio in excess of 10^4^. Barrier height of 0.27 eV was obtained from the temperature dependent I-V characteristics. The heterojunction showed broadband photo-response performance with the maximum external quantum efficiency of 72% from visible to near-infrared range. The maximum photoresponsivity of 475 mA/W and detectivity of 6.5 × 10^11^ Jones was obtained at the wavelength of 860 nm. From the transient properties, rise and decay times were extracted as 19.78 ms and 0.99 ms, respectively. This work shows the potential of sulfurization process for creation of large area MoS_2_ film and its heterojunction formation in highly efficient photovoltaic devices and other applications.

## Figures and Tables

**Table 1:** Comparison of MoS_2_ and Si based photodetectors.

Structure	Method	Responsivity	Detectivity [cm Hz^1/2^/W]	Rise/Decay Time	Reference
MoS_2_/p-Si	Sulfurization	475 mA/W	6.5 × 10^11^	19.78/0.99 ms	This work
MoS_2_/p^+^-Si	Mechanical exfoliation	980 A/W	~10^9^	0.2/2.1 s	[32]
MoS_2_/p-Si	Sulfurization	139 mA/W	N/A	N/A	[14]
MoS_2_/p-Si	Sputtering	~300 mA/W	~10^13^	3/40 μs	[37]

## Supplementary Materials

The following are available online at https://www.mdpi.com/1424-8220/20/24/7340/s1, Figure S1: Raman spectroscopy of the MoO_x_ and MoS_2_ films, Figure S2: Raman spectra of the sulfurized MoS_2_ films on three points, Figure S3: XRD data of the sulfurized MoS_2_ film, Figure S4: XPS data of O 1s core level from MoO_x_, sulfurized MoS_2_, and MoS_2_ bulk crystal, Figure S5: Photocurrent transient characteristics under square pulsed light illumination.
