# Supplementary material for: Photoelectric Characteristics of a Large-Area n-MoS2/p-Si Heterojunction Structure Formed through Sulfurization Process
Source: Sensors (Basel). 2020 Dec 21;20(24):7340. doi: 10.3390/s20247340 (PMC7767020; doi:10.3390/s20247340)
Supplement: Supplementary file 1 [file sensors-20-07340-s001.pdf]

Supplementary Material

Photoelectric characteristics of a large-area n-MoS_2/p-Si heterojunction structure formed through sulfurization process

Yoonsok Kim, Taeyoung Kim, and Eun Kyu Kim*

Department of Physics and Research Institute of Natural Science, Hanyang University, Seoul 04763, South Korea

* *E-mail :* ek-kim@hanyang.ac.kr

Raman spectroscopy

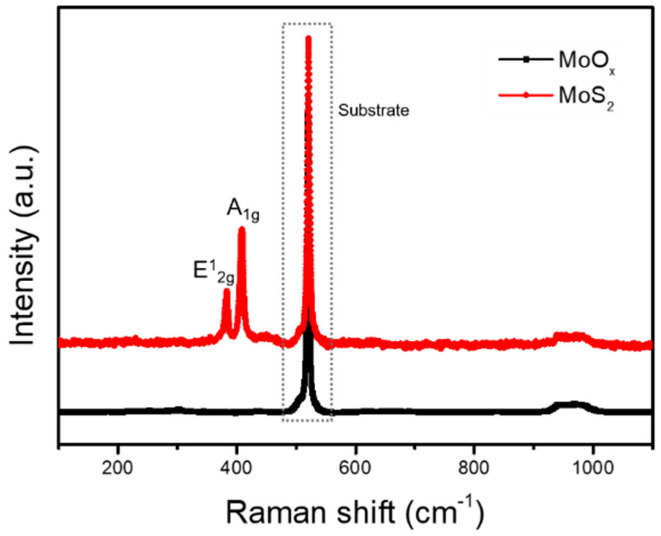

Figure. S1 Raman spectroscopy of the MoO_x and MoS_2 films.

Figure S1. displays Raman spectroscopy results of MoO_x and MoS_2 films in the ranges from 100 to 1100 cm^{-1}. No peaks are observed for thermally evaporated MoO_x film but only substrate peaks. This indicates that the MoO_x film is amorphous structure, same as XRD analysis. This result is consistent with the previous report [1]. While typical two phonon vibration modes were observed on the sulfurized MoS_2 film, E^1_{2g} and A_{1g} phonon vibration mode.

[1] C. Battaglia, X. Yin, M. Zheng, I.D. Sharp, T. Chen, S. McDonnell, Angelica Azcatl, Carlo Carraro, Biwu Ma, Roya Maboudian, Robert. M. Wallace, and Ali Javey, Hole selective MoO_x contact for silicon solar cells, Nano Lett. 14 (2014) 967-971.

Three points of Raman spectra on sulfurized MoS_2 film.

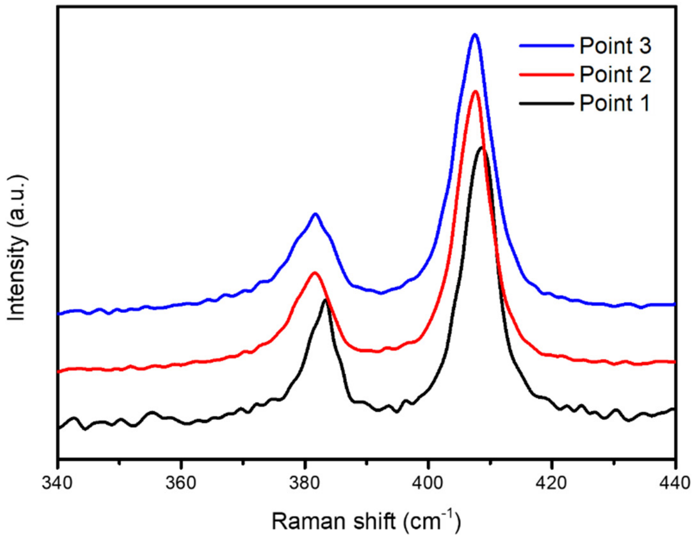

Figure. S2 Raman spectra of the sulfurized MoS_2 films on three points.

Figure S2 shows Raman spectra of randomly measured three random points on the sulfurized MoS_2 film. The data display two phonon vibration modes, E^1_{2g} and A_{1g}, at the same position which indicate that the sulfurized film has homogeneity in the direction of parallel to the surface.

Grain size calculation from XRD profile

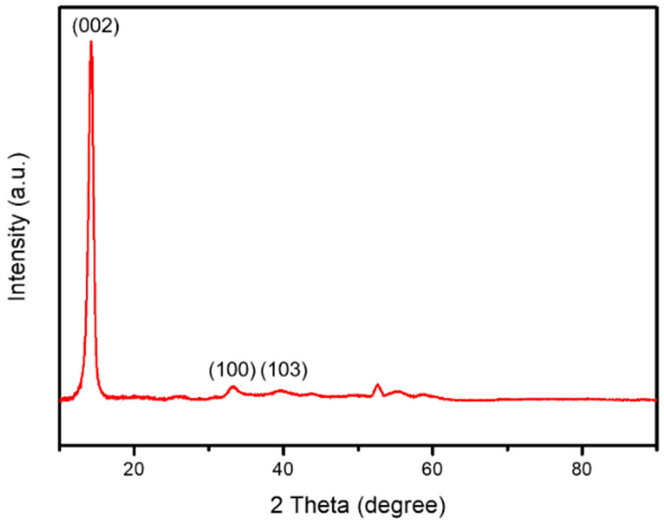

Figure. S3 XRD data of the sulfurized MoS_2 film.

We tried to extract the grain size from the XRD data of the sulfurized MoS_2 film, which have (002)-oriented peak as the main dominant peak, as shown in the Figure S3. The average grain size was extracted to be about 10 nm by Scherrer equation at the (002)-oriented peak.

$$d = \frac{c\lambda}{\beta cos\theta}$$

Here, c is a shape factor taken as 0.9, λ is the X-ray wavelength of Cu K-Alpha (1.5406 Å), β is peak width at FWHM (0.79°), and θ is the peak angle (14.20°).

O 1s core level of XPS peak

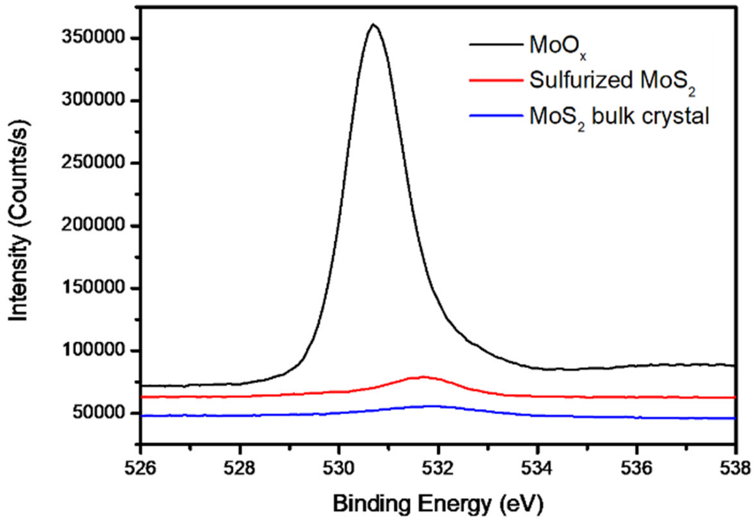

Figure. S4 XPS data of O 1s core level from MoO_x, sulfurized MoS_2, and MoS_2 bulk crystal.

We compared also the XPS data of O 1s core level. Mo:O ratio from MoO_x film, sulfurized MoS_2 film, and MoS_2 bulk crystal appeared 1:2.9, 1:0.3 and 1:0.19, respectively. Also, Mo:S ratio was calculated to be 1:2.4 and 1:2.2 for sulfurized MoS_2 film and MoS_2 bulk crystal, respectively. The Mo:S ratio of the sulfurized film was calculated from the Figure 2c, d. This result indicate that the MoO_x film transformed to MoS_2 film.

Response time measurement

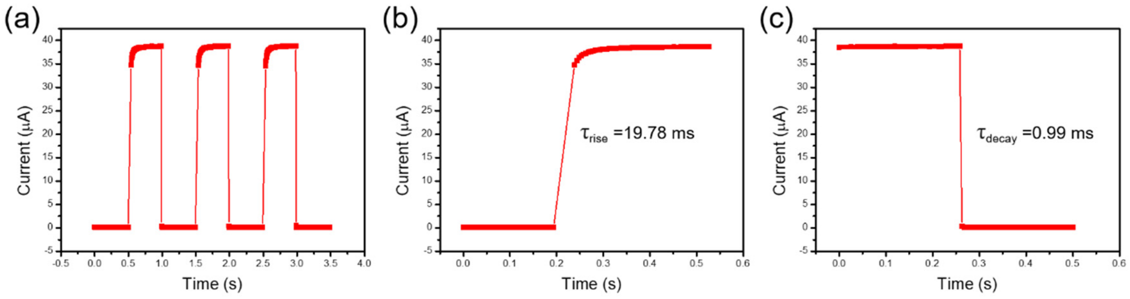

Figure. S5 (a) Photocurrent transient characteristics under square pulsed light illumination of 780nm wavelength at reverse bias of -1 V. (b) The rising and (c) the decaying transients.

Figure S5 represent the response time of the n-MoS_2/p-Si heterojunction. The device shows excellent repeatability and stability. Also, the rise and decay times were calculated to be 19.78 ms and 0.99 ms, respectively.
